# Computer Reconstruction of Plant Growth and Chlorophyll Fluorescence Emission in Three Spatial Dimensions

**DOI:** 10.3390/s120101052

**Published:** 2012-01-18

**Authors:** Chandra Bellasio, Julie Olejníčková, Radek Tesař, David Šebela, Ladislav Nedbal

**Affiliations:** 1 Department of Plant, Soil and Environmental Science, University of Firenze, Viale delle Idee, 30 50019-Sesto Fiorentino, Firenze, Italy; 2 Global Change Research Centre, Academy of Sciences of the Czech Republic, Zámek 136, CZ-37333 Nové Hrady, Czech Republic; E-Mails: olejnickova.j@czechglobe.cz (J.O.); radektesar@gmail.com (R.T.); sebela.d@czechglobe.cz (D.S.); nedbal.l@czechglobe.cz (L.N.); 3 Institute of Physical Biology, University of South Bohemia, Zámek 136, CZ-37333 Nové Hrady, Czech Republic

**Keywords:** 3D reconstruction, chlorophyll fluorescence imaging, leaf area, leaf angle, plant growth, coded light

## Abstract

Plant leaves grow and change their orientation as well their emission of chlorophyll fluorescence in time. All these dynamic plant properties can be semi-automatically monitored by a 3D imaging system that generates plant models by the method of coded light illumination, fluorescence imaging and computer 3D reconstruction. Here, we describe the essentials of the method, as well as the system hardware. We show that the technique can reconstruct, with a high fidelity, the leaf size, the leaf angle and the plant height. The method fails with wilted plants when leaves overlap obscuring their true area. This effect, naturally, also interferes when the method is applied to measure plant growth under water stress. The method is, however, very potent in capturing the plant dynamics under mild stress and without stress. The 3D reconstruction is also highly effective in correcting geometrical factors that distort measurements of chlorophyll fluorescence emission of naturally positioned plant leaves.

## 1. Introduction

Plants continuously acclimate to dynamically changing irradiance, temperature, and humidity [1–6]. This acclimation can involve apparent changes in their three dimensional (3D) structure: e.g., leaf orientation [7–9] and, on a longer scale also, in the size of the leaves as well as their number [10,11]. Plant structure is also the key feature that determines radiative transfer, light capture and light photosynthetic use in the plant canopy, e.g., [12–15]. Modulation in light use efficiency is then reflected in the emission of chlorophyll fluorescence [16–20]. In this paper, we shall simultaneously the two aspects of plant structure and performance. From the plant structure perspective, plant morphology and related traits have been studied in many different ways. A traditional approach usually does not involve the creation of a 3D model of plants [21]. Traits, such as leaf area, leaf angle, ear geometry, are derived from one or more side views of the plants, through algorithms that associate a particular 2D shape to a known parameter.

Methods involving the reconstruction of a 3D plant model can be divided into two categories: passive and active (reviewed by [22]). Passive, less expensive methods include stereo imaging, a method that mimics what happens in human vision [23,24]. Here, 3D reconstruction of the object is based on the use of perspective and difference between two or more images of the object taken from different angles. Then, the 3D model is reconstructed by triangulating the position of points in the two or more different systems of coordinates (matching ‘corresponding points’) [25,26]. Although there are studies reporting the 3D reconstruction of plants using stereovision [23–25,27,28], plants are generally too complex for this method [24,25]. A different approach of creating 3D plant models has been developed by Lemnatech (www.lemnatech.com, Wuerselen, Germany). Here, the 3D plant model is not constructed by aggregating single points, but rather it is carved from a solid volume (for details see www.lemnatech.com, scanalyzer 3D). Pictures of the plant are taken from top view and side views from different angles while the plant is rotated. Areas exceeding the plant outline and ‘non green areas’ within the outline are considered empties and eliminated from each picture. The 3D plant model is then created by merging the two sequences of the ‘carved’ 2D pictures. To date it has been used for modeling plant growth and panicle development of rice and to model plant growth of tomato.

Active methods use an additional light source to illuminate the sample, and include Infra-Red Trianglulation [29], Light Detection and Ranging (LIDAR) [22], and structured light techniques [30,31]. In the recent past, the frequently used LIDAR based on laser scanners have been employed for 3D reconstruction of small plant foliage as well as canopy structure [32,33]. LIDAR technology has also the potential to be combined with other imaging methods, e.g., thermal imaging or chlorophyll fluorescence imaging [22,34–38].

Compared to laser techniques, which illuminate a single point or a section at a time, structured light methods illuminate most of the sample in every picture frame. Structured light is widely used in commercial applications, for example in facial recognition, engineering, arts and architecture (see for example: www.4ddynamics.com; www.accurexmeasure.com; www.gom.com; www.perceptron.com; www.scantech.dk; www.solutionix.com; www.steinbichler.de). But surprisingly, to our best knowledge, it has not been used in Plant Sciences. In structured light techniques the information needed for the reconstruction of the object shape is derived from analyzing the distortion of the light pattern projected on the object, or by triangulation of points (coded light, see below). Generally, the object is illuminated with white light, thus the 3D models of objects having different colors can be reconstructed independently (e.g., fruit and leaves). In addition, structured light techniques do not require movement of the object, so they can be used to study plant growth *in situ*, for example in growth chambers. The reconstructed 3D model can also be combined with other existing imaging instrumentation, giving additional information about the plant metabolism, with an approach similar to the one used by Omasa and coworkers with LIDAR [22,34,35,39].

In this perspective, one of the most powerful optical signals that non-invasively reports on the processes of light capture and photosynthetic efficiency is undoubtedly chlorophyll fluorescence emission (ChlF). The interpretation of ChlF signals has been reviewed in a number of detailed papers and books [16–19,40–44]. The photosynthetic processes are dynamic and heterogeneous in space and so is the ChlF emission [20,27]. The need to capture ChlF emission dynamic heterogeneity led to a development of kinetic ChlF imaging that, since early days [45] and development of the first commercial ChlF imager [27], has become a routine method in Plant Sciences, e.g., [18,28,46–49]. To our best knowledge, the currently available ChlF imagers capture the signal flattened to only two spatial dimensions and time, projecting the third spatial dimension similarly as in conventional photography. In a sky-to-earth perspective, for example, the signals from young leaves at the top of the plants are next to the lower old leaves and the information can only be de-convoluted and classified manually. Similarly so, leaves that are inclined in a sharp angle to the camera axis emit ChlF that is captured in a smaller number of the detecting pixels than the emission from leaves that are perpendicular. Many of necessary corrections of these factors can be done during data processing by human brain. However, fully automated systems capable of capturing plant morphology and fluorescence in growth chambers will improve our understanding of plant responses to stress at the whole plant level, and of the interactions between plants and their microenvironment. Such systems would facilitate the selection of a particular plant phenotype in screening experiments.

The great potential of this approach led us to a construction of a system that performs a highly accurate 3D reconstruction of a plant from multiple real color images and projects the ChlF captured by near-infrared camera on such a reconstruction. We used a particular type of structured light technique, coded light (see Materials and Methods, [50]). In this paper, we report not only on the methods that we have employed to achieve this goal, but also a case study with water stressed young pepper plants.

## 2. Material and Methods

### 2.1. 3D Imaging System Hardware

**Figure 1.**
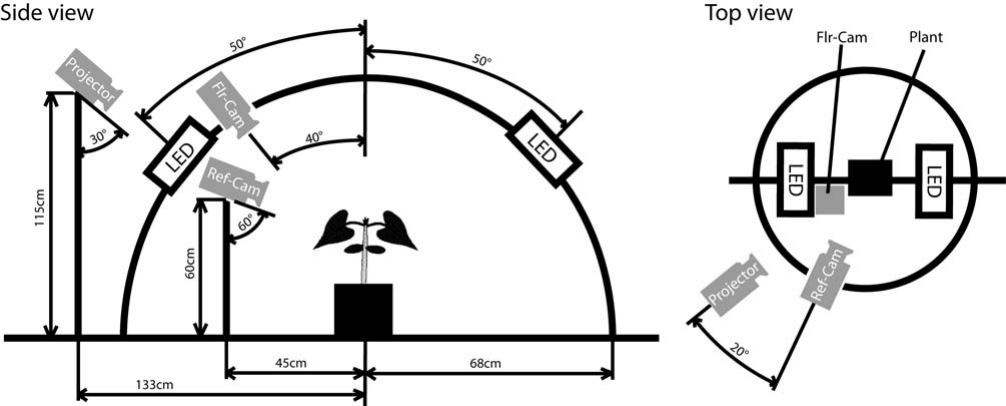
A scheme of the 3D goniometric system. The plant was placed in the center of a circular platform around which the photographic camera rotated at a distance of 0.45 m. The goniometric arch spanning over the plant held two LED (Light Emitting Diode) panels that produced excitation for PAM (Pulse Amplitude Modulation) fluorescence imaging. Also attached to the arch was the fluorescence imaging camera. A DLP projector was used to generate coded illumination on the plant to facilitate the 3D reconstruction.

The 3D imaging was performed using a goniometric plant imaging system developed by Photon Systems Instrument, Ltd. (Brno, Czech Republic, http://www.psi.cz/products/customized-fluorcams/arch-fc-900-a). This system (Figure 1) has been originally designed for ChlF imaging of plants from various angles under a changing angle of irradiation that would mimic the Sun’s movement in the sky.

In order to perform a concurrent 3D reconstruction of plants, a source of coded illumination (a Compaq MP2800 Digital Light Processing projector, 1,024 × 768 pixel screen resolution, Hewlett-Packard Company, Palo Alto, CA, USA) and a reflectance camera (Canon PowerShot G10, Canon Inc., Tokyo, Japan, 5 × Zoom lens–6.1 mm–30.5 mm–F/2.8–4.5, 4,416 × 3,312 pixel resolution of image with 24-bit color depth) were added to the Arch System. Plants were positioned in the center of a circular platform around which the data projector, producing the coded illumination for 3D reconstruction, and the photographic camera were placed. In addition, the Pulse Amplitude Modulation (PAM) fluorescence [51] of the plant was excited by two light-emitting diode (LED) panels and captured by a FluorCam camera (512 × 512 interlaced resolution with 12-bit grayscale depth, Photon Systems Instrument, Ltd., also in Figure 1).

The projector was held 1.15 m above the platform, inclined by 30° down to the plant (Figure 1). The distance between the projector and the center of the imaging platform was 1.33 m. The images of the plant in the coded illumination were captured by the camera (Ref-Cam) held on a stand pole 0.45 m from the platform central rotational axis. The Ref-Cam was 0.6 m above the platform inclined by 60° down towards the plant. In the simple protocol used here, the plant was photographed in this way from two opposite perspective angles of 0° and 180°. For more complex canopies, combinations of four or more viewing angles could be applied. Such a configuration can be reached by permanent placement of 2–4 cameras at appropriate angles and distances from the plant.

The system described here was used to take two separate sequences of pictures: the changes of the ChlF distribution under actinic irradiance exposure (ChlF kinetic imaging) and the sequence of plant photographs necessary for 3D reconstruction. The latter is composed of a plant picture in full light and 48 plant pictures in coded light. From the combination of these 48 images, each point of the plant can be unequivocally identified, since it is illuminated by a unique light/dark code consisting of 48 ones (light) or zeros (dark). This unique light/dark code is used for identification the 3D coordinates of each plant point (see Section 2.1.2). Then, the 3D plant model can be calculated from a cloud of such 3D points that were identified as belonging to the plant (see Section 2.1.3. 3D Reconstruction).

#### 2.1.1. Cameras and Projector Calibration

The camera calibration determines which light beam of the real world is captured by each pixel of the image. In other words the calibration assigns an equation to each captured pixel and turns each captured light beam into a ‘camera light vector’ with a known equation. The equation describes the coordinates of the 3D space captured by each pixel of the camera. Such a calibration was performed on both Ref/Cam and FluorCam cameras. It was achieved using two perpendicular chessboards (one horizontal, one vertical) that were placed in the center of the imaging platform facing cameras. The underlying method has been described by Tsai [52]. The horizontal chessboard had 8 × 10 squares whereas the vertical one had 7 × 11 squares. Every square was 0.026 × 0.026 m.

The Ref-Cam as well as the FluorCam were focused at the center of the circular imaging platform and the focus remained stable for the entire experiment. The images of the two perpendicular chessboards were analyzed to identify semi-automatically the edges of all the chessboards squares (according to “Camera Calibration Toolbox for Matlab”, http://www.vision.caltech.edu/bouguetj/calib_doc/index.html). The exact knowledge of the real world coordinates of the squares together with the localization of the corresponding points in the captured photographic image yielded the required calibration in the form of a projection matrix [53]. In this way, both the photographic camera and the FluorCam were calibrated.

The calibration of the projector was performed similarly. Here, the calibration assigned an equation to each light beam projected on the plant, describing the coordinates of the illuminated 3D space. Each pixel of the projector was then transformed in a ‘projector light vector’ with a known equation. Calibration was performed by projecting a chessboard image on a white projection plane that was consecutively moved to three different horizontal levels: 0 m, 0.5 m, and 1 m above the imaging platform. The projected chessboard image consisted of 8 × 10 squares, each of 50 × 50 pixels. The real world coordinates of the projected squares were determined and this knowledge was combined with the coordinates from the projected image to yield the projector calibration [53].

#### 2.1.2. Identification of the 3D Points Coordinates—Pairing of Camera and Projector Vectors

Crucial steps in the 3D reconstruction are pairing the projector light vector with the RefCam light vector for each point of the plant, and triangulating the vectors to retrieve the 3D coordinates of the plant point. The traditional approach involved the identification of corresponding points in a pair of stereoimages. The matching operation is hard with plants that usually do not have sufficiently contrasting features, veins or edges in their leaves, and in fact it requires manual matching [24]. An alternative approach to automate the “matching of corresponding points” is called coded light and has been described by Altschuler [50]. In the coded light technique one of the two cameras used in stereoimaging is substituted by a projector. The projector, instead of acquiring the information relative to the plant points, projects the information to the plant, which is then acquired by the camera. In this linear flow, the information needed for the triangulation of points (*i.e.*, the knowledge of the two light vector equations) is completely delivered to the camera and no matching is needed [30,54].

Briefly, the light sequence that is projected on the plant is designed in such a way that each pixel of the projector delivers a unique sequence of illumination with a defined light pattern (light code). In this way each illuminated point of the plant carries the information relative to the projector light vector that illuminate it.

**Figure 2.**
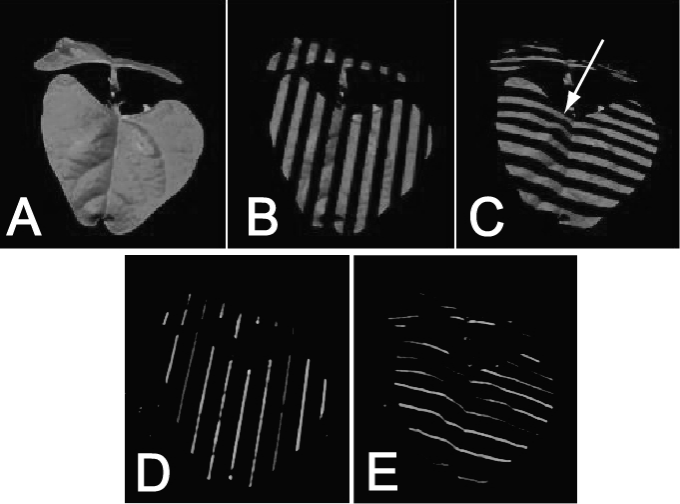
Coded light illumination of a *Phaseolus vulgaris* plant. Panel (**A**) shows full illumination. Panel (**B**) and (**C**) show pictures extracted from the Gray code illumination pattern, panel (**D**) and (**E**) show picture extracted from the Phase Shift Sequence illumination patterns. In total 48 pictures were taken in coded light plus a picture in full light. Since the pictures are taken with exactly the same settings, they can be overlapped. Each pixel will be characterized by a sequence of null and ones (dark and illuminated) that identifies individually the light beam that was shined on the captured point.

The projected pattern used here contained two different codes: a Gray code and Phase Shift Sequence (for details see [55]). The Gray code consisted of projected sequence of stripes of varying thickness (Figure 2(B,C)) projected first horizontally, then vertically. The coarsest projected pattern was half black, half white. The second coarsest pattern was a quarter black, a quarter white, a quarter black, and a quarter white. The further narrowing of the white and black stripes continued until the stripes were only eight projector pixels wide, *i.e.*, we used eight patterns in one direction and eight patterns in the perpendicular direction, yielding in total 16 images, all with relatively coarse illumination patterns.

To explore the resolution limits of the employed approach, we also used fine projector patterns (Phase Shift Sequence). The Phase Shift Sequence consisted of 1-pixel wide white lines that were intervened with 15-pixel wide black stripes (Figure 2(D,E)). Sixteen such images were projected. They differed in the relative positions of the white stripes that were shifted by one pixel in the consecutive imaging steps. The same operation was repeated for the perpendicular orientation of the stripes, so we obtained 32 plant images in the Phase Shift Sequence illumination pattern. Thus, the coded light sequence was formed by 48 pictures in total: 16 plant pictures in Gray code illumination patterns (Figure 2(B,C)) and 32 plant pictures in Phase Shift Sequence illumination patterns (Figure 2(D,E)).

In these pictures, each pixel appears either illuminated or dark depending on position and illumination pattern. Each pixel was, characterized by a vector of 48 nulls or ones after assigning illuminated pixel 1 and dark pixel 0 in the 48 pictures sequence. The position of the pixel in the picture informed about the camera light vector, while the 48 bit binary illumination code informed about the projector vector that illuminated the point. Pairing of these vectors has been used to identify unique 3D points of the sample (see Section 2.1.3).

#### 2.1.3. 3D Reconstruction

During the 3D imaging one picture of the plant under white light and 48 pictures under coded light were captured (Figure 2). In order to exclude pixels not belonging to the modeled plant, green pixels, representing the green parts of the modeled plant, were extracted from the RGB picture taken in in full light (Figure 2(A)). Such a filtered picture has been overlapped with the sequence of 48 pictures with the projected light pattern and only those pixels identified as belonging to the plant were extracted from the set of 48 pictures and used for 3D reconstruction. The position of the pixel in the plant picture informed about the camera light vector, while the 48 bit binary illumination code informed about the projector vector illuminating the point (see Section 2.1.2). In such a way each pixel was unique and independent since it was characterized by a unique vector of 48 zeroes or ones (dark or illuminated). By matching the plant photograph with 48 bit illumination code we were able to determine by triangulation the real world coordinates of every green points. The 3D position of each point was calculated independently [55,56].

In order to reduce noise in the 3D reconstruction, we excluded isolated points that had less than 10 other points in their vicinity (that was defined by a sphere of 5 mm diameter). This selection criterion was identified empirically; points that were more than 5 mm apart frequently originated in the empty space, e.g., between two neighboring leaves. Each point in the 3D space identified as belonging to the plant was connected to the two closest neighbor plant points creating a triangle in the Delaunay method [57,58]. The 3D reconstruction of the plant surface was, thus, generated from such elemental triangles.

**Figure 3.**
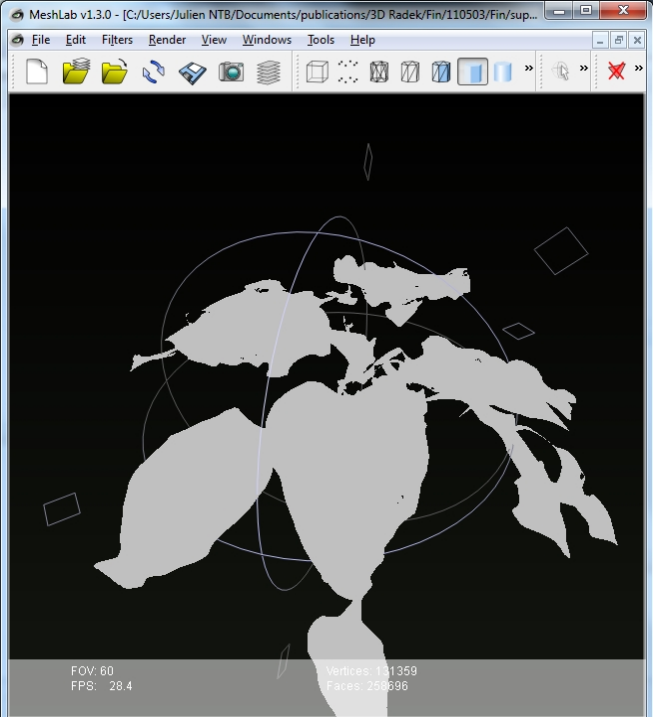
3D point reconstruction of a pepper plant visualized by Meshlab open source software (http://meshlab.sourceforge.net/).

By this process, 3D plant reconstruction of the investigated plant photographed from one side was achieved. Then, the process was repeated from the other side of the plant rotated by 180°. The two reconstructions were merged together to include all the plant surfaces that may have been obscured in the one side imaging (Figure 3). More complex plant canopies may require imaging from more than two sides.

### 2.2. Chlorophyll Fluorescence Emission

Chlorophyll a fluorescence (ChlF) of the plant was excited by two panels of light-emitting diodes (LEDs, λ_max_ ≈ 635 nm), as shown in Figure 1. The LED panels generated both measuring light flashes as well as actinic irradiance as described by Nedbal *et al.* [27]. The measuring protocol started by a 20 min dark adaptation of the plant kept in the center of the goniometric platform. Then, the plant was exposed to actinic irradiance light (100 μmol(photons) m^−2^·s^−1^ for 120 s eliciting a fluorescence transient that was captured by FluorCam in series of 512 × 512 pixel images of 12-bit grayscale depth). The fluorescence emission was imaged by the FluorCam camera using sparse measuring flashes and PAM detection [51]. The ChlF images of plant were taken 50 times per second. For simplicity, we have used here only two images: the image of the peak fluorescence emission (Fp) that was typically observed around one second into the period of actinic light exposure and the image of the steady-state emission (Fs) that reached after ∼120 s of actinic irradiance. These two images were combined into a single image using the formula for plant vitality index of the photosynthetic apparatus Rfd [59], which is used as a non-destructive indicator of the photosynthetic rate. Since in our case the ChlF was induced by very low actinic light, we labeled the fluorescence index Rfd100. The calculation of the fluorescence index was following: Rfd100 = (Fp − Fs)/Fs, where number 100 indicated the intensity of actinic light. Rfd100 images were used to compare the plants response to low irradiance exposure and just to roughly estimate the photosynthetic efficiency of the leaves.

### 2.3. Modulation of the Fluorescence Signal by Geometrical Factors

The ChlF emission recorded by the FluorCam is proportional to the leaf area imaged in a camera pixel. This area increases with the square of the distance between the leaf and the camera. However, ChlF decreases with the square of that distance. These two effects, which often dominate the distance dependence of the imaged ChlF signal, cancel each other. Hence, fluorescence signals recorded in a camera pixel will not depend strongly on the distance between the FluorCam and the leaf.

On the other hand, the ChlF emission depends on the orientation of the leaf to the camera. Assuming isotropic fluorescence emission from a leaf surface and isotropic excitation irradiance, the ChlF signal in a camera pixel is proportional to the fluorescent surface area imaged in a camera pixel. Concretely, the camera pixel captures ChlF from an elemental leaf surface S/cos(ϕ), where S is the area imaged by the pixel in perpendicular orientation and ϕ is the angle between the camera optical axes and the leaf normal. Thus, the signal detected in FluorCam increases with the increasing leaf surface angle so that, e.g., for 45° leaf inclination the detected signal is higher by 41% than with perpendicular orientation (here we assume that re-absorption of the ChlF in the leaf tissue is negligible). This is approximately the case with largely isotropic irradiance generated in FluorCam with multiple LED panels of different angles of light incidence to the leaf surface. In another case when the exciting irradiance is anisotropic, the leaf angle dependence of the FluorCam signal may be even stronger than with the isotropic irradiance, e.g., when the angle of irradiance is largely different from the FluorCam optical axis. In contrast, the leaf angle dependence of the FluorCam signal can be negligible when the exciting irradiance comes anisotropically from the side of the FluorCam—then, the incident photon flux density is proportional to cosine of the angle between the leaf normal and the camera/LED axis. That effect cancels 1/cosine angle dependence of the signal detection.

In either case, one can use the ChlF emission mapped on the 3D reconstruction of the investigated plant (see Section 3.2 Chlorophyll Fluorescence Measurements) to perform the necessary geometric corrections of the signal. Also, the 3D information on the distribution of ChlF can be used to perform proper averaging of the fluorescence signal over the plant surface—a task hardly possible without 3D reconstruction.

### 2.4. Plants

The 3D reconstruction performance was evaluated in three types of experiments using different potted plants bought from a local garden store. The capacity to properly capture widely diverse morphological features was evaluated by using beans, cycad, and hibiscus, plants that differ greatly for leaf morphology and architecture. The capacity to capture growth dynamics as well as the impact of drought stress was evaluated on young pepper plants (*Capsicum annuum L.*). Pepper plants ∼120 mm high, were acclimated for two weeks in a growth chamber (SGC 170PHX-J, Sanyo Gallenkamp, The Hague, The Netherlands). Growth conditions were: 16 hour day, 25 °C, 40% humidity and PAR (photosynthetically active radiation) of 250 μmol (photons) m^−2^·s^−1^, followed by 8 hour dark, 20 °C, and 60% humidity. During acclimation plants were watered daily. Afterwards, 14 pepper plants were divided into four groups labeled HW, HS, LW, LS. The HW and HS plants (three repetitions) were exposed in the growth chamber to high irradiance of 450 μmol (photons) m^−2^·s^−1^ of PAR. The LW and LS (four repetitions) were in low irradiance of 85 μmol (photons) m^−2^·s^−1^ of PAR. Before the experiment the total water pot capacity was quantitated by drying a set of three pots in a drying oven at 80 °C until constant weight was reached. During the experiment, HW and LW plants were watered to 100% water pot capacity, while HS and LS plants were watered to 30% of pot capacity.

The imaging procedure was performed on HW, HS, LW, and LS plants, two hours after the light was switched on in the morning. The optical measurements included photographing the plants in coded illumination and ChlF imaging, as described above. As a reference, the ChlF was also measured directly on the 5th and the 6th leaves by the conventional imaging FluorCam. The reference imaging was performed with an identical protocol to the measurement described in Section 2.2. For a geometrical reference, we also measured the height of each plant by a ruler. At the end of the experiment, plants were scanned to determine their true leaf area with imageJ (http://rsbweb.nih.gov/ij/) as described in [60].

## 3. Results and Discussion

### 3.1. Method Development and Validation

**Figure 4.**
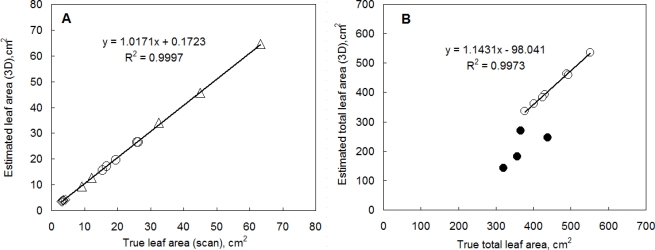
Comparison of direct measurement of plant leaf area with the area determined from the 3D computer reconstruction. **(A)** Measurement of individual leaves that differed in their size and morphology (bean [⋄], cycad [○] and hibiscus [Δ]). The leaves were attached to plants in their natural position. **(B)** Measurement of total leaf area of whole pepper plants that differed in their cultivation conditions: well watered plants cultivated in high and low light (450 and 85 μmol (photons) m^−2^·s^−1^ of photosynthetically active radiation, respectively) [○] and water stressed plants in high light [•]. The lines represent a linear regression optimized by Microsoft Excel.

The leaf area and leaf angle calculated from a 3D reconstruction are features of high relevance that can be easily verified by direct measurements. In order to test the accuracy of the 3D reconstruction, we reconstructed individual leaves of several plant species that differed in their leaf size and morphology (bean, cycad, hibiscus). Leaves were left attached to the plant in their natural position. Figure 4(A) shows a high correlation between the leaf area determined by 3D imaging system and the scanned leaf area. The leaf size was between 3 and 65 cm^2^. The 3D reconstruction yielded high fidelity estimates.

We have also reconstructed the total leaf area of young pepper plants (Figure 4(B)). The 3D reconstruction determined the leaf area with an accuracy of 97% in the case of well-watered plants. Leaf area of low light water stressed plants (open symbols in Figure 4(B)) was estimated by a similar accuracy since the plant were fully turgid. The total leaf area of the plants was between 35 cm^2^ and 70 cm^2^. In the case of high light water stressed plants the reconstruction significantly underestimated the total leaf area (closed symbols in Figure 4(B)) because plants were wilted and a significant overlap of some leaves obscured part of the leaf area in imaging.

**Figure 5.**
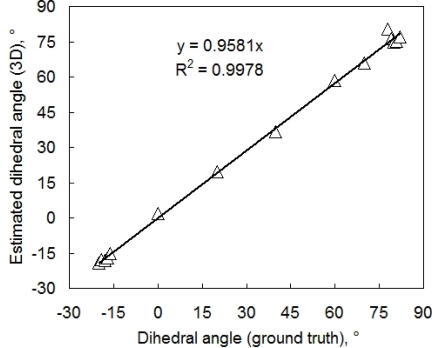
Validation of measurements of dihedral angle between a leaf that was positioned horizontally and a leaf that was precisely rotated. The dihedral angle between the two leaves was first measured and adjusted using a water-level inclinometer (x-axis). The measured angles are compared with the angles obtained by imaging and 3D computer reconstruction (y-axis). The negative and positive angles differ by the direction of the leaf rotation. The line represents a linear regression optimized by Microsoft Excel.

Leaf angle is another important parameter. To evaluate the accuracy of leaf inclination estimate we cut two leaves from a bean plant and glued them to two flat supports. The first leaf was placed in a horizontal position near the center of 3D imaging system. The second leaf was placed nearby on a adjustable holder so that the angle between the leaf and RefCam camera axis could be modified. For each chosen angle, the two leaves were imaged and the dihedral angle between the two leaves was determined from the resulting 3D reconstruction. The reference measurement was performed using a water-level inclinometer (with a precision of approximately 1°). The correlation between reference and reconstructed angle is shown in Figure 5.

**Figure 6.**
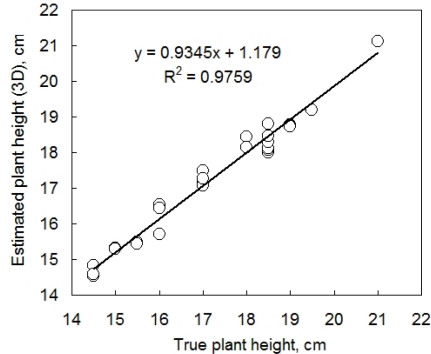
Validation of measurements of plant height. Twelve young pepper plants were measured daily for five consecutive days. The interpolating line represents a linear regression optimized by Microsoft Excel.

Further, we tested the accuracy of plant height determination in the 3D computer reconstruction (Figure 6). The plant height was calculated as a position of the highest point of plant 3D reconstruction minus the pot height. The true plant height was measured by a ruler. These two measurements were compared daily on 14 individual pepper plants during five consecutive growth days. Both plant height and leaf angle were precisely estimated by 3D reconstruction. Figure 6 informs also about the precision of the measurement: the computer reconstruction was able to resolve clusters of plant heights that were indistinguishable with the ruler. In fact measuring the plant with a ruler disturbs the position of the leaves and required rounding to the closest 5 mm, while the 3D reconstruction is able of measuring precisely the plant without any disturbance.

**Figure 7.**
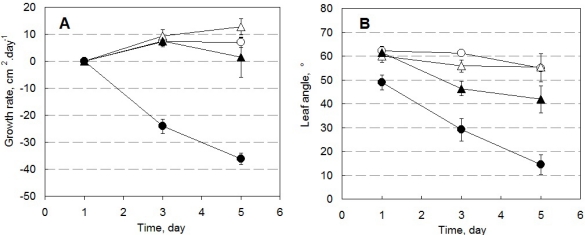
Growth rate and leaf angle measurement by 3D reconstructions. (**A**) The growth rate measured by expansion (positive values) or reduction (negative values) of integral plant leaf area per day during five consecutive days. (**B**) The angle between the camera optical axis and the leaf plane. The wilting in the high light drought stressed plants (HS) and, partially, also in the low light drought stressed plants (LS) is reflected in a decreased leaf angle. Pepper plants were cultivated for five days in following conditions: HW ([○], 450 μmol (photons) m^−2^·s^−1^ of PAR, 100% pot water capacity), HS ([•], 450 μmol (photons) m^−2^·s^−1^ of PAR, 30% pot water capacity), LW ([Δ], 85 μmol (photons) m^−2^·s^−1^ of PAR, 100% pot water capacity), and LS conditions ([▴], 85 μmol (photons) m^−2^·s^−1^ of PAR, 30% pot water capacity). Symbols represent mean values of three plant measurements ± standard deviation.

Pepper plants in HW, HS, LW, and LS conditions differed in their growth rate, leaf angle as well as in ChlF emission. Figure 7 shows growth rates calculated from the change of total leaf area determined by the 3D imaging system (Figure 7(A)), and from the change of leaf angle (angle between the plane passing through the leaf and the vertical axis) during 5 days of the experiment (Figure 7(B)). The negative growth rate shown in Figure 7(A) for drought-stressed plants in high light is a result of leaf overlap that obscures some leaf area in the 3D reconstruction.

### 3.2. Chlorophyll Fluorescence Measurements

The ChlF emission intensity is proportional to the absorbed photosynthetically active radiation (PAR) multiplied by the ChlF quantum yield. The ChlF intensity is thus modified by the distance from the light source as well as by the position, particularly by the angle of the leaf toward the camera. The fluorescence emission is usually considered to be isotropic in all directions, a property that can be modulated by significant re-absorption in the direction approaching tangent of the emitting leaf surface.

The ChlF yield may vary rapidly in time due to competition of the emission with highly dynamic primary photosynthetic charge separation in Photosystem II (photochemical quenching) as well as due to photoprotective non-photochemical quenching of fluorescence emission [43]. On a longer scale, ChlF is also influenced by leaf age, pigment composition or by various stress factors [59,61–64]. For example, fluorescence measurements of whole plants can be more affected by leaf age or the proportion of veins and lamina than by the angle effect [64]. For a proper interpretation of the ChlF emitted from plant leaves positioned at different angles and distances relative to the camera, we projected the captured ChlF emission over the plant 3D reconstruction (*3D ChlF imaging*) of young pepper plants with different water status and grown under two irradiation levels.

**Figure 8.**
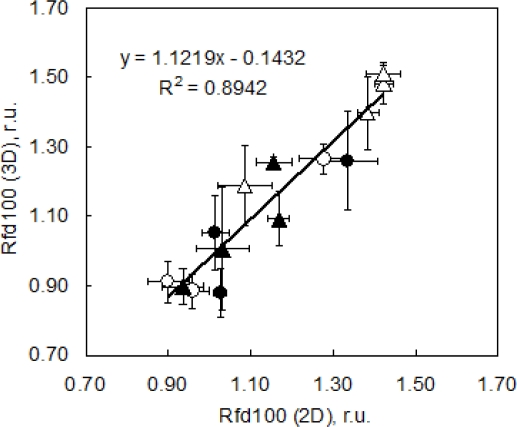
Correlation between Rfd100 parameter measured under low actinic light exposure (100 μmol (photons) m^−2^·s^−1^, 120 s) by conventional 2D imaging fluorometer FluorCam and by projection of fluorescence emission on 3D computer reconstruction of plants. The data points represent well watered plants in high light (HW, [○], 450 μmol (photons) m^−2^·s^−1^ of PAR, 100% pot water capacity), water stressed plants in high light (HS, [•] 450 μmol (photons) m^−2^·s^−1^ of PAR, 30% pot water capacity), well watered in low light (LW, [Δ] 85 μmol (photons) m^−2^·s^−1^ of PAR, 100% pot water capacity), water stressed plants in low light (LS, [▴] 85 μmol (photons) m^−2^·s^−1^ of PAR, 30% pot water capacity), r.u. stands for relative units.

As a suitable ChlF parameter we chose to image Rfd100 (= (Fp − Fs)/Fs, where Fp is fluorescence at peak P and Fs is for fluorescence at the steady state level under 100 μmol (photons) m^−2^·s^−1^). Rfd100 can be recorded without application of saturating pulse that may be difficult to achieve in complex plant canopies and in automated high-throughput experiments. Rfd100 was chosen to serve the purpose of the presented case study: to show the capabilities of *3D ChlF imaging*. We propose that a different experimental setup might be appropriate for a more complex physiological screening. Figure 8 shows a correlation of the Rfd100 index measured in the goniometer setup (Figure 1) and projected on the 3D reconstruction of pepper plants with reference measurements done using a FluorCam conventional imaging fluorometer. Both measurements were done on the same leaves, *i.e.*, on 5th and 6th leaves of each measured plants. Symbols represent means of five repetitions, and error bars show standard deviations. Symbols differentiate between high light acclimated plants that were well watered (open circles) and drought stressed (closed circles) and low light acclimated plants that were well watered (open triangles) and those that were drought stressed (closed triangles).

After five days of cultivation, high light plants expressed lower Rfd100 values than low light plants (HW = 0.96 ± 0.05, HS = 1.03 ± 0.06, LW = 1.42 ± 0.09, LS = 1.16 ± 0.08) most probably because they had lower chlorophyll content (data not shown) and were acclimated to a five times more intense irradiation. In this case LW and LS plants displayed a well-known shade acclimation pattern, with higher chlorophyll content, due to their larger antenna size [48]. Low light plants thus coped better with low light irradiance during ChlF measurements than high light plants. We are aware that Rfd measured under very low light cannot be fully correlated with CO_2_ assimilation rate. The resulting low Rfd100 values reflect only the state of the small portion of the chloroplasts that receive light [65–67], however, it can be used to compare the plants response under low irradiance exposure.

**Figure 9.**
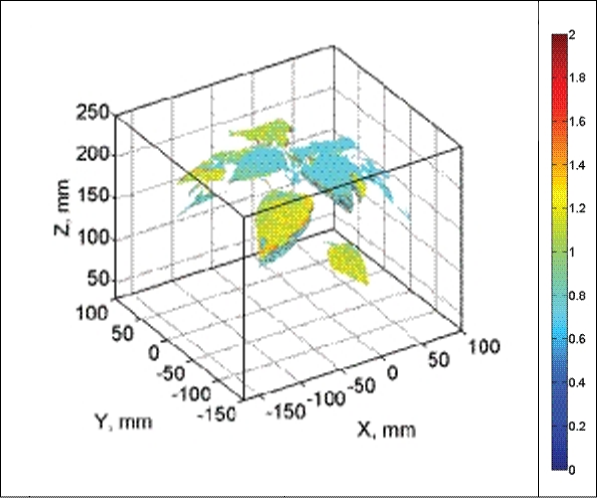
Distribution of Rfd100 parameter over water stressed pepper plants cultivated for five days in low light conditions (LS: 85 μmol (photons) m^−2^·s^−1^, 30% pot water capacity). Rfd100 parameters were measured by projection of chlorophyll fluorescence emission on 3D computer reconstruction of plants.

HW, HS and LW plants displayed no heterogeneity in Rfd100 distribution over the whole plants meaning that all parts of the plant had identical response to the low actinic light exposure. Sole LS plants showed clear vertical distribution of Rfd100; leaves on the top expressed lower Rfd100 and lower leaves expressed higher Rfd100 values (Figure 9). We propose that this vertical distribution is a signature of beginning water stress that is first manifested in top leaves that have higher transpiration rates. We propose that such 3D imaging of ChlF can provide additional valuable information about the plant metabolism over the whole plant body as shown by this case study.

**Figure 10.**
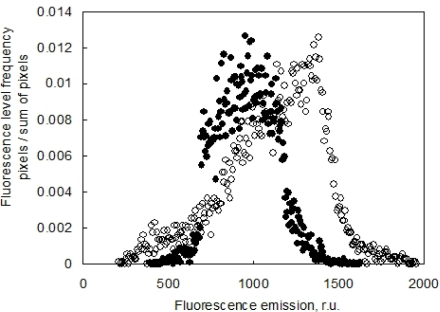
Distribution of steady state (Fs) chlorophyll fluorescence emission of a pepper leaf. Open symbols [○] show the distribution of fluorescence as recorded by FluorCam image while closed symbols represent the same values corrected for the leaf inclination [•]. The 3D corrected data were calculated by renormalizing the fluorescence signal by cosine of the angle between the normal to the leaf surface and the optical axes of the camera.

It is important to note that the advantage of combining the ChlF imaging with 3D computer reconstruction is a correct re-normalization of the signal to the leaf angle. In Section 2.3 we discussed the angle effect on ChlF signal captured by the camera. The effect of leaf angle is demonstrated in Figure 10, where the histogram of Fs signal obtained before (in standard 2D imaging) and after the re-normalization (3D reconstruction of the fluorescence emission) is presented. The difference between the uncorrected (open symbols) and corrected fluorescence signal (closed symbols) is due to an average leaf declination of 38° of the particular pepper leaf. The difference is even significantly larger in wilted leaves.

### 3.3. Advantage of the Coded Light Approach

There are some particular features that make the coded light approach interesting for plant research. It is an affordable method: good results can be achieved with off-the-shelf consumer electronics. Plants can be studied from a static point of view, even from above. The algorithms used can be computed without dedicated software. Imaging does not need any movement of the object and the 3D model can be also combined with other existing imaging instrumentation giving additional information about the plant metabolism. These features make coded light ideal for studying plant growth and physiology in a growth chamber using a built-in system that automatically observes plant growth from above. Such a fully automated system can facilitate the phenotype selection in screening experiments and will improve the understanding responses to stress and of interactions between plants and their microenvironment. In such studies ChlF imaging can be utilised as a proxy for understanding spatio-temporal effects of stress at the whole plant level. Since plants are illuminated with white light and imaged in RGB, additional information could be retrieved without adding complexity to the imaging and calculation system. In this study only the green channel was used for studying plant morphology, but similarly a 3D model of fruit could be constructed selecting a different colour channel. This feature opens up a wide array of additional possibilities, e.g., the study of senescence, flower development, fruit ripening, *etc*.

## 4. Conclusions

We conclude that it is feasible to reconstruct the plant morphology in three spatial dimensions using coded light and to capture plant growth. The correction of chlorophyll fluorescence signals to reconstructed plant morphology has a significant impact on proper interpretation of the captured signals. Without 3D reconstruction, an increase in recorded chlorophyll fluorescence emission that is due to a leaf angle change by wilting can be misinterpreted as an increase of the emission yield. We conclude that 3D computer reconstruction of plant morphology combined with chlorophyll fluorescence imaging has a strong potential in automated high-throughput phenotyping of plants.

## Abbreviations

ChlF: Chlorophyll a fluorescence
Fp: Peak fluorescence emission observed after ∼1 second of actinic light exposure
Fs: Steady-state fluorescence emission in light-adapted state measured after 120 seconds of actinic light
LED: Light-emitting diode
PAM: Pulse Amplitude Modulation
PAR: Photosynthetically active radiation
Rfd100: Fluorescence index measured under low actinic irradiation (100 μmol (photons) m^−2^·s^−1^) calculated as follows: Rfd100 = (Fp − Fs)/Fs
3D: Three spatial dimensions
